# Deciding what is possible and impossible following hippocampal damage in humans

**DOI:** 10.1002/hipo.22694

**Published:** 2017-01-07

**Authors:** Cornelia McCormick, Clive R. Rosenthal, Thomas D. Miller, Eleanor A. Maguire

**Affiliations:** ^1^ Wellcome Trust Centre for NeuroimagingUniversity College LondonLondonUnited Kingdom; ^2^ Nuffield Department of Clinical NeurosciencesUniversity of OxfordOxfordUnited Kingdom

**Keywords:** impossible scenes, scene construction, amnesia, semantic knowledge, hippocampus

## Abstract

There is currently much debate about whether the precise role of the hippocampus in scene processing is predominantly constructive, perceptual, or mnemonic. Here, we developed a novel experimental paradigm designed to control for general perceptual and mnemonic demands, thus enabling us to specifically vary the requirement for constructive processing. We tested the ability of patients with selective bilateral hippocampal damage and matched control participants to detect either semantic (e.g., an elephant with butterflies for ears) or constructive (e.g., an endless staircase) violations in realistic images of scenes. Thus, scenes could be semantically or constructively ‘possible’ or ‘impossible’. Importantly, general perceptual and memory requirements were similar for both types of scene. We found that the patients performed comparably to control participants when deciding whether scenes were semantically possible or impossible, but were selectively impaired at judging if scenes were constructively possible or impossible. Post‐task debriefing indicated that control participants constructed flexible mental representations of the scenes in order to make constructive judgements, whereas the patients were more constrained and typically focused on specific fragments of the scenes, with little indication of having constructed internal scene models. These results suggest that one contribution the hippocampus makes to scene processing is to construct internal representations of spatially coherent scenes, which may be vital for modelling the world during both perception and memory recall. © 2016 The Authors. Hippocampus Published by Wiley Periodicals, Inc.

## INTRODUCTION

If we close our eyes, we can readily construct vivid scenes in our mind's eye that are spatially coherent and richly detailed in semantic content. Such scenes feature prominently when we recall past experiences, imagine fictitious or future events, and even when we plan routes during navigation. Bilateral lesions to the hippocampi in humans impair all of these abilities (Scoville and Milner, 1957; Maguire et al., 2006; Hassabis et al., 2007; Mullally et al., 2012). Interestingly, even the capacity to discriminate between visual scenes that are in plain sight seems to be compromised in these patients (Lee et al., 2005a, 2005b; Aly et al., 2013), suggesting that scene perception may also require the hippocampus (see also Mullally et al., 2012; Zeidman and Maguire, 2016). This constellation of findings has been interpreted in different ways (Lee et al., 2005a, 2005b; Shrager et al., 2006; Hassabis and Maguire, 2007; Kim et al., 2011; Aly et al., 2013; Maguire and Mullally, 2013; Zeidman and Maguire, 2016). Consequently, there are different views about the precise role played by the hippocampus in scene processing.

One account posits that a primary function of the hippocampus is to construct internal models of the world in the form of spatially‐coherent scenes (Hassabis and Maguire, 2007; Maguire and Mullally, 2013; Zeidman and Maguire, 2016). This scene construction system can be driven ‘offline’ during imagination and memory recall, while also continually constructing and refining a representation of the scene currently being experienced ‘online’ during perception (Mullally et al., 2012; Aly et al., 2013). A consequent prediction of this account is that hippocampal‐damaged patients should be impaired at selecting a target scene from among distractor images of a different scene that are shown from slightly different angles. This is because making such a discrimination judgement necessitates the internal modelling of the scenes to arbitrate between the given options. Patients do indeed show this scene discrimination deficit (Lee et al., 2005a, 2005b). Overall, therefore, according to the scene construction account of the hippocampus, whenever modelling of a scene is necessary or advantageous – anywhere across cognition, and this includes functions such as perception, decision‐making, as well as memory and navigation – the hippocampal scene construction process will be engaged and deficits will be apparent in hippocampal‐damaged patients (Mullally and Maguire, 2014; Zeidman and Maguire, 2016).

Undoubtedly, however, patients with bilateral hippocampal damage do not display frank perceptual problems, and the most striking feature of their neuropsychological profile is an episodic long‐term memory deficit (Scoville and Milner, 1957; Penfield and Milner, 1958). Another account of the hippocampus therefore argues that its role is fundamentally mnemonic (Squire, 1992). According to this view, the scene discrimination deficits described above are interpreted not as a scene perception impairment but due instead to the behavioural tasks exceeding the limited capacity of short‐term memory, thereby engaging long‐term memory. Hence, the patients, with their long‐term memory deficit, are unable to hold the information relating to one scene in memory to compare it to other scenes in a stimulus array (Shrager et al., 2006; Kim et al., 2011). Moreover, proponents of this view have failed to find impaired mental construction of fictitious and future scenes in hippocampal‐damaged patients (Squire et al., 2010; Kim et al., 2015), although such deficits have now been widely reported (Maguire and Mullally, 2013; reviewed in Clark and Maguire, 2016) and methodological issues may explain the null findings (Maguire and Hassabis, 2011; Maguire et al., 2015).

**Figure 1:**
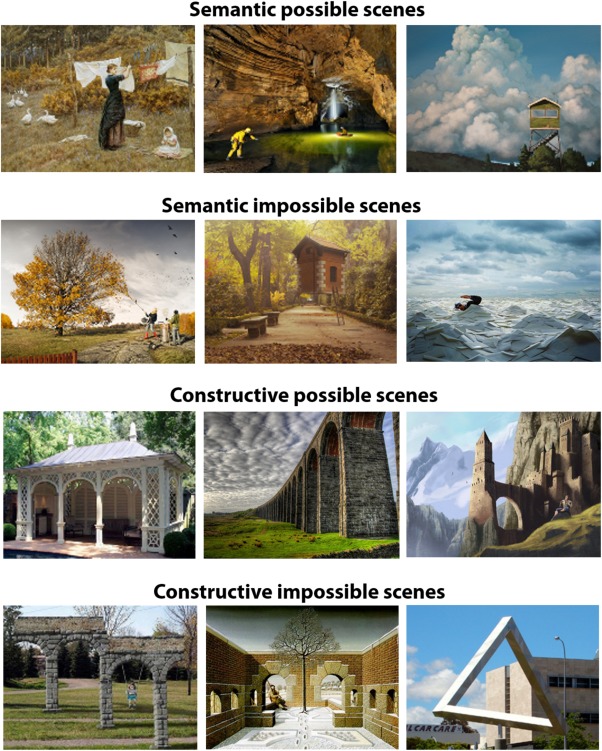
Example stimuli. Semantic scenes are presented in the the upper two panels: the possible semantic scene depicts a woman hanging up some laundry, whereas the impossible semantic scene below shows a woman vacuuming the leaves from a tree, which would not happen in the real world. The lower two panels depict examples of constructive scenes. On the left side of the panel, a possible constructive scene includes a typical pavilion, whereas an impossible constructive scene beneath shows arches that would not be possible to build in the real world. In particular, the top connecting structure suggests a flat architecture, the columns of the arches are located at different depths within the scene. Impossible pictures were adapted from the following sources: Semantic: http://www.erikjohanssonphoto.com/; http://www.ucreative.com/inspiration/surreal-photography-of-flying-house-by-rafa-zubiria/; http://www.gettyimages.co.uk/detail/photo/businessman-swimming-in-sea-of-envelopes-high-res-stock-photography/200354836-001; Constructive: http://www.moillusions.com/funny-lookin-arch-illusion/; http://impossible.info/english/art/mey/mey3.html ; https://upload.wikimedia.org/wikipedia/commons/3/38/Perth_Impossible_Triangle.jpg. [Color figure can be viewed at wileyonlinelibrary.com]

Hence, the current debate revolves around the question of whether the hippocampus' contribution to scene processing is constructive, perceptual or mnemonic. To disambiguate these accounts, we tested patients with selective bilateral hippocampal damage on a new task designed to control for general perceptual, mnemonic and basic task demands, meaning that we could isolate the requirement to internally construct spatially coherent representations of scenes. We were inspired by mathematical artists such as Penrose and Escher who created images depicting impossible spatial constructions, such as the famous endless staircase (Penrose and Penrose, 1958; Cowan, 1974; Cowan, 1977; Kulpa, 1987; Schacter et al., 1995; Lee and Rudebeck, 2010; Douglas et al., 2016). Importantly, in our experiment, every individual part of a scene was spatially coherent, but holistically the image challenged the fundamental spatial construction of real‐world scenes (Fig. 1). We reasoned, and confirmed in a pilot study, that to discriminate between possible and impossible constructive scenes, one has to construct an internal model of an intact scene and then match and compare that model to the perceived scene. Thus, by having participants decide whether an image was constructively possible or impossible, we were able to probe the scene construction process with high specificity. We also included a control condition involving semantic possible and impossible scenes––for example, an elephant with butterfly ears, or vacuuming a tree (see Fig. 1). We confirmed in our pilot study that the distinction between possible and impossible semantic scenes required participants to look at the image, understand relationships between the semantic elements of the scene, and make a decision about its semantic connotation. Importantly, the spatial constructive aspect of these scenes was normal, with only the content violating semantic knowledge of what is possible in the real world. Furthermore, since the task involved viewing and making a decision about one scene at a time, with the scene always visible to the participant, we eliminated the need to compare two or more images as in previous scene discrimination studies and thereby excluded demands on long‐term memory (Lee et al., 2005a, 2005b). Overall, therefore, we reasoned that the general perceptual and mnemonic demands were held constant across both constructive and semantic conditions, because all of the stimuli were similar images of realistic scenes and participants were asked to make a possible/impossible decision after every image.

**Figure 2:**
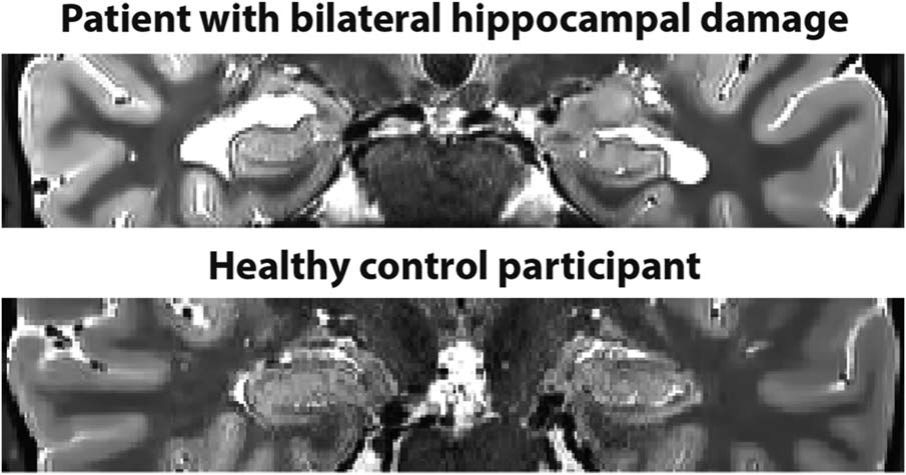
Characterization of hippocampal damage. Example T2‐weighted structural MR image of a patient with selective bilateral hippocampal damage (upper panel) and an age, gender and IQ‐matched healthy control participant (lower panel). Images are displayed in native space corresponding approximately to the position of *y* = −10 in the MNI coordinate system.

We hypothesised that if the contribution of the hippocampus to scene processing is inherently constructive (Zeidman and Maguire, 2016), then the patients would have difficulty processing the spatial‐constructive aspects of scenes and consequently would be selectively impaired at discriminating between possible and impossible scenes only in the constructive condition. An implication of such a result is that scene discrimination and scene construction deficits in patients with bilateral hippocampal damage could be driven by a spatial‐constructive rather than a general scene encoding or mnemonic impairment.

## MATERIALS AND METHODS

### Participants

Six patients [all right‐handed males, mean age 57.0 (SD 16.9) years, age range: 27–70] with selective bilateral hippocampal lesions and selective episodic memory impairment took part (see Tables 1 and 2 for demographic information and neuropsychological profiles). Hippocampal damage resulted in all cases from voltage‐gated potassium channel (VGKC)‐complex antibody‐mediated limbic encephalitis (LE). In line with previous reports of this patient population (Dalmau and Rosenfeld, 2014), manual (blinded) segmentation of the hippocampi from high‐resolution structural MRI scans confirmed that our patients showed volume loss confined to the left [Patients – HC: 2506 mm^3^ (mean) ±394 (standard deviation), control participants – CTL: 3173 mm^3^ ±339, *t*(15) = 3.7, *P* = 0.002, Cohen's *d* = 1.8] and right hippocampus [HC: 2678 mm^3^ ±528, CTL: 3286 mm^3^ ±301, *t*(15) = 3.1, *P* = 0.008, Cohen's *d* = 1.4]. To rule out gross differences between patients and controls elsewhere in the brain, an automated voxel‐based‐morphometry (VBM, Ashburner, 2009) analysis was carried out using voxel‐by‐voxel comparisons on whole brain T1 weighted MRI images (for imaging details see Callaghan et al., 2015). There were no differences in grey matter volume between the groups outside of the hippocampus, even at a liberal uncorrected *P*‐value of less than 0.001 and cluster threshold of 50 voxels. Neuropsychologically, the patients displayed an impairment in immediate and delayed memory recall, and they recollected significantly fewer episodic, but not semantic, details on the Autobiographical Interview (Levine et al., 2002), as detailed in Table 2.

**Table 1:** Summary of Demographic Information

Group	N	HD	Age	Chronicity	LHC vol*	RHC vol*
HC group	6 (M)	6 (R)	57.0	6.8	**2506**	**2678**
			16.9	2.1	394	528
CTL group	12 (M)	11 (R)	57.2	n.a.	**3173**	**3286**
			16.6		339	301
*P* value			0.98	n.a.	**0.002**	**0.008**

For both groups, means are displayed with standard deviations underneath. HC, hippocampal‐damaged patients; CTL, healthy control participants; M, Male; HD, Handedness; n.a., not applicable; R, Right; L, Left; vol, volume in mm^3^. *One control participant could not be scanned, therefore HC volumes are based on all six patients and 11 control participants. Age and chronicity are described in years. *P*‐value = *P*‐value of two‐sample *t*‐test with significant differences depicted in bold.

**Table 2:** Summary of Neuropsychological Profile

	WASI‐M	WASI‐S	AMint*	AMext*	IRM	DRM	RM	SEM	WM	Lang	EF	Perc	Mood
**HC**	13.2	12.8	**31.7**	6.1	**−0.7**	**−0.7**	**−**0.3	**−**0.3	**−**0.3	**−**0.3	**−**0.2	**−**0.4	0.0
	2.2	1.8	6.7	3.8	0.8	0.8	1.1	1.2	0.8	0.9	0.5	1.6	1.0
**CTL**	13.8	11.8	**51.3**	5.9	**0.3**	**0.4**	0.1	0.1	0.1	0.1	0.1	0.2	0.0
	1.5	2.6	13.6	2.2	0.3	0.6	0.6	0.9	1.1	0.9	0.6	0.3	0.8
*P*	0.46	0.41	**0.01**	0.92	**0.001**	**0.01**	0.29	0.39	0.42	0.37	0.39	0.22	0.94

For both groups, means are displayed with standard deviations underneath. HC = hippocampal‐damaged patients; CTL = healthy control participants; *P* = *P*‐value of two‐sample *t*‐test with significant differences (all memory‐related) depicted in bold. The WASI‐M and WASI‐S are shown as scaled score means, and the Autobiographical Interview scores are shown as standard means for this test. The other scores (where available scaled scores) of individual tests have been transformed into *z*‐scores and averaged across patients and controls within each neuropsychological domain. Therefore, a mean *z*‐score of zero indicates that both groups had the same mean. Domains contained the following subtests: WASI‐M = Matrix Reasoning and WASI‐S= Similarities subtest of the Wechsler Abbreviated Scale of Intelligence (WASI; Wechsler, 1999). AM = autobiographical memory interview (Levine et al., 2002): int = internal (episodic) details, ext = external (semantic) details. *Of note, autobiographical memory performance of the patients was compared to a separate control group (5 males, 1 female, mean age 55.2 ± 18 years, range 22–69, all right‐handed). IRM = immediate recall memory: Wechsler Memory Scale (WMS‐III; Wechsler, 1997), logical memory 1 units and thematic scores, wordlist 1 total recall, and Rey‐Osterrieth complex figure immediate recall (Osterrieth, 1944). DRM = delayed recall memory: WMS‐III logical memory 2 units and thematic scores, and Rey‐Osterrieth complex figure delayed recall. RM = recognition memory: Warrington Recognition Memory Test for words and faces (Warrington, 1984), WMS‐III wordlist 2 recognition. SEM = semantic memory: Warrington Graded Naming Test (McKenna and Warrington, 1980; Warrington, 2010). WM = working memory: WMS‐III digit span subtest. Lang = language abilities: Delis‐Kaplan Executive Function System (D‐KEFS) letter fluency and category fluency tests (Delis et al., 2001). EF = executive functions: D‐KEFS category switch test, word‐colour interference test, trails test (average of visual scanning, number sequencing, letter sequencing, number‐letter switching, and motor speed tests), Hayling Test Sentence Completion Test (Burgess and Shallice, 1997). Perc = perception: Visual Object and Space Perception Battery (VOSP) dot counting, cube analysis, position discrimination tests (Warrington and James, 1991), and the Rey‐Osterrieth Complex Figure copy. Mood = Hospital Anxiety and Depression Scale (HADS; Zigmond and Snaith, 1983).

Twelve healthy control participants also took part [all male, one left‐handed, mean age 57.2 (16.6) years, age range from 25 to 77]. There were no significant differences between patients and controls on age, general cognitive ability and a range of neuropsychological tests assessing semantic memory, language, perception, executive functions and mood (see Table 2). In addition to comparing the two groups overall, we ensured that each patient was matched closely to two of the control subjects on sex, age, and general cognitive ability. All participants gave informed written consent in accordance with the local research ethics committees.

### Imaging Details

#### High resolution T2‐weighted structural MRI scans of the medial temporal lobes

Five of the patients and 10 of the control participants underwent structural MR imaging limited to a partial volume focused on the temporal lobes using a 3.0‐T whole body MR scanner (Magnetom TIM Trio, Siemens Healthcare, Erlangen, Germany) operated with a radiofrequency (RF) transmit body coil and 32‐channel head RF receive coil. These structural images were collected using a single‐slab 3D T2‐weighted turbo spin echo sequence with variable flip angles (SPACE, see Mugler et al., 2000) in combination with parallel imaging, to simultaneously achieve a high image resolution of ∼500 μm, high sampling efficiency and short scan time while maintaining a sufficient signal‐to‐noise ratio (SNR). After excitation of a single axial slab the image was read out with the following parameters: resolution = 0.52 × 0.52 × 0.5 mm^3^, matrix = 384 × 328, partitions = 104, partition thickness = 0.5 mm, partition oversampling = 15.4%, field of view = 200 × 171 mm 2, TE = 353 ms, TR = 3200 ms, GRAPPA × 2 in phase‐encoding (PE) direction, bandwidth = 434 Hz/pixel, echo spacing = 4.98 ms, turbo factor in PE direction = 177, echo train duration = 881. K‐space averaging was employed to boost SNR with 90% resampling (i.e., average factor 1.9) weighted to the centre of k‐space. For reduction of signal bias due to, for example, spatial variation in coil sensitivity profiles, the images were normalized using a prescan, and a weak intensity filter was applied as implemented by the scanner's manufacturer. It took 12 min to obtain a scan.

#### High resolution T1‐weighted structural MRI scans of the whole brain at 3.0 tesla

In addition, five of the patients and 11 of the control participants underwent a whole brain structural T1weighted sequence at an isotropic resolution of 800 µm (Callaghan et al., 2015) which was used for the automated VBM analysis (one control participant could not be scanned). These images had a FoV of 256 mm head‐foot, 224 mm anterior‐posterior (AP), and 166 mm right‐left (RL). This sequence was a spoiled multi‐echo 3D fast low angle shot (FLASH) acquisition with a flip angle of 21° and a repetition time (TR) of 25 ms. To accelerate the data acquisition, partially parallel imaging using the GRAPPA algorithm was employed in each phase‐encoded direction (AP and RL) with forty reference lines and a speed up factor of two. Gradient echoes were acquired with alternating readout polarity at eight equidistant echo times ranging from 2.34 to 18.44 ms in steps of 2.30 ms using a readout bandwidth of 488 Hz/pixel (Helms and Dechent, 2009). The first six echoes were averaged to increase SNR (Helms and Dechent, 2009) producing a T1‐weighted image with an effective echo time of 8.3 ms.

#### High resolution T1‐weighted MRI scan of the whole brain at 7.0 tesla

One patient could not be scanned at our Centre due to recent dental implants. We therefore used images acquired previously on a 7.0 Tesla MRI scanner––a three‐dimensional whole‐brain T1‐ weighted phase sensitive inversion recovery sequence (Mougin et al., 2015) with 0.6 × 0.6 × 0.6 mm^3^ resolution with a tailored inversion pulse for magnetization inversion at ultrahigh field (Hurley et al., 2010), which provided inherent bias field correction.

#### Hippocampal segmentation

To improve the SNR of the anatomical images, two or three T2‐weighted high resolution scans were acquired for a participant. Images from each participant were co‐registered and denoised following the Rician noise estimation (Coupe et al., 2010). The denoised images were averaged and smoothed with a full‐width at half maximum kernel of 2 × 2 × 2 mm. In each case, left and right hippocampi were manually (blindly) segmented and volumes extracted using the ITK Snap software version 3.4.0 (Yushkevich et al., 2006).

#### VBM analysis

An automated VBM analysis was performed using SPM12 (Statistical Parametric Mapping, Wellcome Trust Centre for Neuroimaging, London, UK). The averaged T1‐weighted images were segmented into grey and white matter probability maps using the unified segmentation approach (Ashburner and Friston, 2005). Inter‐subject registration of the tissue classes was performed using Dartel, a nonlinear diffeomorphic algorithm (Ashburner, 2007). The resulting Dartel template and deformations were used to normalize the tissue probability maps to the stereotactic space defined by the Montreal Neurological Institute (MNI) template. For VBM analysis, the normalization procedure included modulating the grey matter tissue probability maps by the Jacobian determinants of the deformation field and smoothing with an isotropic Gaussian smoothing kernel of 8 mm full width at half maximum (FWHM). The normalised grey matter images from controls and the patients with hippocampal damage were contrasted in a voxel‐by‐voxel manner using a two sample t‐test and thresholded at *P* < 0.001 uncorrected and a cluster extend of 50 voxels.

### Stimuli

The images for the main experiment were closely matched between conditions in their format (horizontal: 450 pixels (high) × 600 pixels (wide), vertical: 600 × 450 pixels; on average 10 vertical images per condition, range from 8 to 12) and whether they were photographs or paintings (on average 13.5 paintings per condition, range from 12 to 14). All images were in colour except for two (one semantic possible and one semantic impossible scene). The content of the images was carefully matched across semantic and constructive images (e.g., a possible and an impossible semantic landscape or a possible and an impossible constructive tower). However, we ensured, via pilot testing, that participants were not aware of this.

### Task Procedure

Before the main experiment, participants underwent a practice session. They were told that they would be viewing pictures of scenes on a computer screen one at a time and that they should look very carefully at these pictures because some of the scenes would depict something that is not possible. Each condition was explained separately in detail using hard copies of example images. In the first instance, semantic and constructive violations were pointed out to the participant and great care was taken to ensure that participants understood what was meant by these errors. That is, for the semantic violations, the participants were instructed to check whether the content of an image looked right to them (e.g., an elephant with butterfly ears, flying on clouds, breathing under water). For constructive violations, participants were instructed to check whether the image depicted a spatially implausible scene (e.g., wrong perspectives, endless staircases). In addition, various descriptions of the term “impossible” (e.g., “not quite right,” “odd,” “highly unlikely”) were incorporated throughout the instructions and practice session to ensure that participants understood the concept. During the task, participants were presented with one scene image at a time and were simply asked to decide whether they thought the current scene depicted something that was possible or impossible in the real world and to indicate their response via a key press. Participants were not explicitly told whether a picture belonged to the semantic or constructive condition. Following each possible/impossible decision, they were asked to rate how difficult they found it to decide whether a scene was possible or impossible, and then how confident they were in their decision.

Following these instructions, participants completed a practice session on the computer. There were eight images (two per condition) in the practice session. The experiment was run using Cogent 2000 version 125 (Wellcome Trust Centre for Neuroimaging and Institute of Cognitive Neuroscience, UCL, London, UK). Each image was presented for three seconds at the centre of the screen before the question “Is this scene possible or impossible? 1––possible, 3––impossible” appeared underneath it. Participants then had up to an additional 15 s to look at the scene image and question and indicate their decision by pressing either key number 1 (possible) or 3 (impossible). After participants responded, the scene image disappeared and the difficulty question and its rating scale (1 = not at all, 2 = somewhat, 3 = very) appeared on the screen. Once the difficulty rating was made, the confidence question and its rating scale (1 = not at all, 2 = somewhat, 3 = very) appeared on the screen. Participants were then prompted to press the space bar to proceed to the next scene image. For both difficulty and confidence ratings, participants had a maximum of 15 s to respond before continuing onto the next trial. During the practice session, the experimenter also provided verbal feedback for each image. If there were any mistakes in assigning an image to either possible or impossible, the experimenter would bring up the image on the computer screen again after completion of the practice session and explain the difference between both categories again for each of the mistakes until the participant comprehended the task instructions.

On completion of the practice session, the participants completed two blocks of the main task, each containing 50 images. The images were presented in pseudo random order so that no more than two images of the same condition were presented consecutively. The timings of the main experiment were identical to the practice session. Completion of the practice and main experiment took participants ∼40 min.

### Debriefing

To explore any potential group differences in strategies used during the task, we asked each participant the following debriefing questions immediately; that is, less than a minute after completing the task:

1. How did you do the task? Did you have a strategy for how you made up your mind whether a scene was possible or not?
2. What was your general mind set in the experiment? How did you approach each scene? (Here, we aimed to further explore the strategies used.)
3. Did you know any of the images from before the experiment? (All participants answered no to this question.)

Patients and control participants were able to give detailed and insightful responses to these questions.

### Data Analysis

Kolmogorov‐Smirnov tests confirmed that the data were normally distributed. We therefore used separate two‐way repeated measures analysis of variance (2 way‐RM‐ANOVA) with participant group (patients, control participants) as a between subject factor with two levels and scene category as a repeated measurement (within subject) factor with four levels (possible semantic, impossible semantic, possible constructive and impossible constructive) to assess significance levels of hit rate, reaction times and rating responses. Main effects and interaction effects were evaluated first, and a two‐sided *P*‐value of less than 0.05 was used to reject the null hypothesis in each case. Where there were significant main or interaction effects, all possible post‐hoc comparisons between groups and scene categories were conducted using Sidak's multiple comparison tests, again using a two‐sided corrected *P*‐value of less than 0.05.

Pairwise independent comparisons between both groups (e.g., discrimination scores, hippocampal volumes) were conducted using Student's two sample *t*‐test. Again, a two‐sided threshold of *P* less than 0.05 was considered statistically significant. To examine potential group by stimulus interactions, we conducted Pearson's correlations on the hit rate per stimulus between controls and patients for all scene images, and separately for each scene category. A two‐sided *P*‐value of less than 0.05 was again considered statistically significant.

To enhance the interpretability of the results and where appropriate, we also report the effect sizes (using Cohen's *d*) and show the individual data from each participant.

## RESULTS

### Discrimination

**Figure 3:**
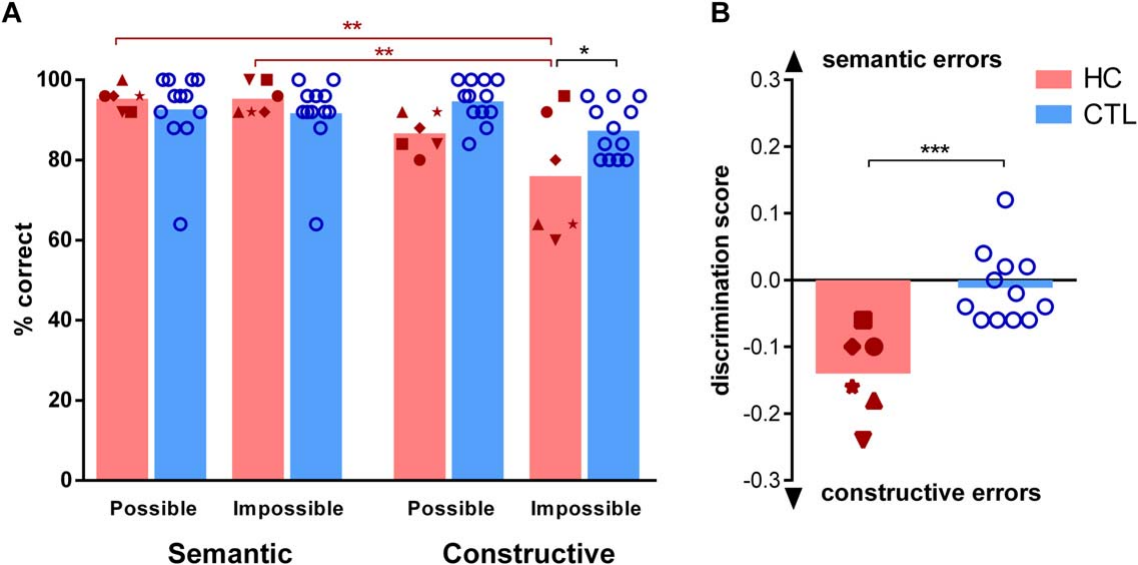
Task results. **A**: Percent accuracy for each condition for individual patients with hippocampal damage is shown (HC, red symbols, *n* = 6) and healthy control participants (CTL, blue circles, *n* = 12). The height of the bars represents the mean. **=*P* < 0.01; ***=*P* < 0.001. Between‐group effects are indicated in black, within group effects are indicated in colour (HC in red). Hippocampal damaged patients were selectively impaired in detecting constructive impossible scenes. **B**: The dissociation between semantic and constructive impossibility detection is shown. The discrimination score is defined as the difference between correctly classified constructive and correctly classified semantic scenes divided by the maximal number of correct answers in a category. A maximum score of 1 indicates only semantic errors with no misclassified constructive scenes and a minimum score of −1 indicates only constructive errors with no misclassified semantic scenes. Controls misclassified approximately the same amount of semantic and constructive scenes (hence a discrimination score around zero), whereas patients with hippocampal damage made significantly more errors on the constructive scenes (and hence have a negative discrimination score). [Color figure can be viewed at wileyonlinelibrary.com]

Examining the accuracy of all four scene conditions (semantic possible, semantic impossible, constructive possible, and constructive impossible), we found a significant main effect of scene category [*F*(3,48) = 7.7, *P* = 0.0003] and an interaction effect between participant group (patients/healthy control participants) and scene category [*F*(3,48) = 3.3, *P* = 0.027] whereas the main effect of group was not significant [*F*(1,16) = 2.5, *P* = 0.13; see Figure 3a for individual performance scores and Table 3 for means and standard deviations for each scene category]. Patients with hippocampal damage performed at a similar high level of accuracy as the control participants when making judgements about the semantic possible [Sidak's post hoc test, *t*(64) = 0.6, *P* = 0.95, Cohen's *d* = 0.3], semantic impossible [*t*(64) = 0.9, *P* = 0.85, Cohen's *d* = 0.5], and constructive possible [*t*(64) = 1.94, *P* = 0.21, Cohen's *d* = 1.6] scenes. By contrast, patients identified significantly fewer constructive impossible scenes than control participants [*t*(64) = 2.7, *P* = 0.03, Cohen's *d* = 0.9]. Moreover, patients categorized significantly fewer constructive impossible scenes correctly than they did semantic possible [*t*(48) = 4.1, *P* = 0.022, Cohen's *d* = 1.7] and impossible scenes [*t*(48) = 4.1, *p* = 0.022, Cohen's *d* = 1.7]. No other post hoc comparison within the patient or the control group or between groups revealed a significant result, indicating that the observed effect was specific to the patients' categorisation of impossible constructive scenes.

**Table 3:** Summary of Behavioural Results on the Impossible Scenes Task

Group	Scene category	Accuracy		RT (sec)		Difficulty*		Confidence*	
**HC**	Semantic possible	95.3	*3.0*	4.2	*2.1*	1.2	*0.2*	2.8	*0.2*
	Semantic impossible	95.3	*3.9*	2.9	*1.5*	1.1	*0.1*	2.8	*0.1*
	Constructive possible	86.7	*4.8*	4.6	*1.6*	1.3	*0.2*	2.7	*0.2*
	Constructive impossible	76.0	*15.6*	4.0	*1.3*	1.2	*0.2*	2.8	*0.2*
**CTL**	Semantic possible	92.7	*10.1*	4.0	*2.4*	1.3	*0.2*	2.8	*0.2*
	Semantic impossible	91.7	*9.4*	3.4	*2.3*	1.2	*0.3*	2.8	*0.2*
	Constructive possible	94.7	*5.2*	4.3	*3.1*	1.5	*0.3*	2.7	*0.2*
	Constructive impossible	87.3	*6.8*	3.9	*1.8*	1.4	*0.2*	2.7	*0.2*
2way‐RM‐ANOVA		**sig.**		n.s.		n.s.		n.s.	

For both groups, means are displayed with standard deviations in italics to the right. HC = hippocampal‐damaged patients; CTL = healthy control participants; 2way‐RM‐ANOVA = 2‐way‐repeated‐measures Analysis of Variance; sig = significant main effect of scene category and interaction effect (for exact statistics, see main text); n.s.=no significant main or interaction effects; Accuracy displayed as percent hit rate; RT = reaction times, calculated from the onset of the ‘possible/impossible’ question; sec = seconds; Difficulty and confidence ratings ranged from 1 (not at all) to 3 (very); *=one patient rated both difficulty and confidence for every picture at level 3, we therefore excluded his ratings.

Making judgements between possible and impossible scenes that are either semantic or constructive is essentially asking the same question––to know what is impossible, one has to know what is possible. Hence, we calculated a discrimination score for each participant, defined as the difference between all constructive scenes correctly classified and all semantic scenes correctly classified, divided by 50 (the total number of semantic or constructive images). A value of zero therefore indicates an equal number of errors for semantic and constructive scenes. A negative score indicates more errors for constructive scenes (with a minimum of −1) and a positive score indicates more errors for semantic scenes (with a maximum of 1). Using this discrimination score, the difference between patients with hippocampal damage and controls on this task became very evident (Fig. 3b). In comparison to control participants, patients made significantly more discrimination errors for constructive than semantic scenes [Patients: −0.14 (mean) ±0.07, Controls −0.01 ±0.05, 2‐sided *t*‐test, *t*(16) = 4.4, *P* = 0.0005, Cohen's *d* = 2.1].

### Other Task Parameters

**Figure 4:**
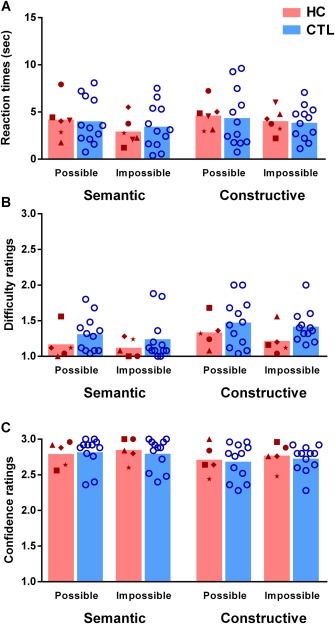
Reaction times and ratings. **A**: Reaction times (calculated from the onset of the ‘possible/impossible’ question) are shown in seconds (bar indicates the mean) for the possible/impossible decision of individual patients (HC red symbols) and control participants (CTL blue circles). There were no significant differences between conditions or groups. **B**: Difficulty ratings where the bar indicates the mean, 1 = very easy…3 = very difficult. Note that difficulty and confidence rating data from one patient were excluded – see text. There were no significant differences between conditions or groups. **C.** Confidence ratings where the bar indicates the mean, 1 = not confident at all…3 = very confident. There were no significant differences between conditions or groups. [Color figure can be viewed at wileyonlinelibrary.com]

Reaction times for all scene categories were similar for control participants and patients [*F*(1,16) = 0.0008, *P* = 0.978, see Fig. 4 for individual data points and Table 3 for means and standard deviations]. Following each possible/impossible decision, participants were asked about the difficulty of this discrimination. Of note, one participant rated all scenes as maximally difficult (response key 3) and maximal confident (response key 3). We therefore excluded his difficulty and confidence rating responses from the analysis. None of the 2way‐RM‐ANOVAs revealed any significant main or interaction effects. Most important for our study, both subject groups rated difficulty as equally low, regardless of whether scenes were semantic or constructive, [*F*(1,15) = 3.1, *P* = 0.097]. Moreover, when asked to rate their confidence in their possible/impossible decision, both groups expressed high confidence across scene categories [*F*(1,15) = 0.05, *P* = 0.82].

Considered with the scene discrimination results, these findings indicate that patients with hippocampal damage did not process the constructive scenes as accurately as control participants but were generally unaware of this deficit, since they did not rate constructive scenes as being more difficult to judge, and retained high confidence in their decisions.

### Group by Stimulus Interactions

We next considered whether the significant accuracy result was in any way influenced by patients responding in a different manner to the stimuli compared to control participants. We conducted correlation analyses between the number of times a scene was correctly identified as possible or impossible by patients and controls. That is, if the pattern of responses to the scene stimuli was different between the groups (e.g., if patients randomly selected possible and impossible), we would expect no correlation of correct responses between the groups. However, correct responses, collapsed across scene categories, correlated significantly between patients and control participants (100 scene images, Pearson's *r* = 0.52, *R* ^2^ = 0.28, *P* < 0.0001). This finding indicates that scene images that were classified correctly by control participants, were also classified correctly by the patients, and similarly for those stimuli that were erroneously classified. When analysed as a separate subgroups, this correlation was also significant for the constructive (50 scenes, *r* = 0.58, *R* ^2^ = 0.33, *P* < 0.0001) and semantic (50 scenes, *r* = 0.46, *R* ^2^ = 0.21, *P* = 0.0009) images. Hence, the response profile to individual stimuli did not differ between patients and control participants; it was just that the patients were significantly poorer at discriminating between possible and impossible constructive scenes.

### Qualitative Exploration of Strategies

**Figure 5:**
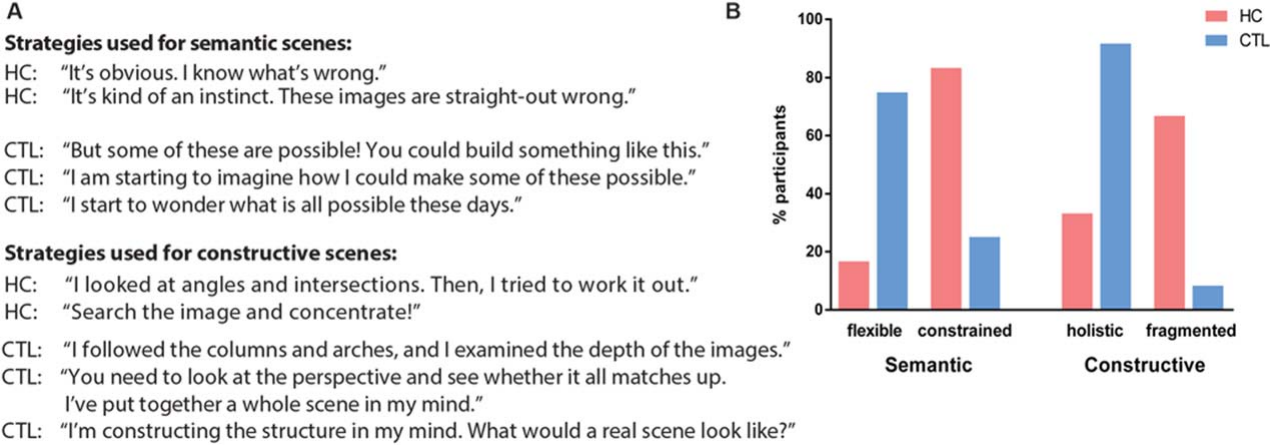
Exploration of task‐related strategies. **A**: Examples of the strategies for detecting semantic and constructive impossible scenes as described by patients with hippocampal damage (HC) and healthy control participants (CTL). **B**: Strategies expressed as the percentage of participants (patients in red and healthy control participants in blue), who used the strategy. For semantic scenes, the majority of patients described a constrained and abstract focus on the semantic content of an image, whereas the majority of controls additionally engaged in manipulation of image content flexibly and creatively in their mind's eye. For constructive scenes, the patients typically focused on specific fragments of an image, whereas controls constructed an internal spatially coherent representation of the entire scene. [Color figure can be viewed at wileyonlinelibrary.com]

To explore any potential group differences in the strategies that were used to make the possible/impossible decision for each category of scene, participants were asked a series of open‐ended questions immediately after the conclusion of the task. Interestingly, the responses for both semantic and constructive scenes differed considerably between the patients and control participants (Fig. 5).

For the semantic scenes, five of the patients but only three of the control participants responded that they knew instantaneously whether an image was right or wrong, that it was a quick and constrained process. However, nine control participants, but only one patient spontaneously explored the scenes and thought creatively and flexibly about how an impossible scene could be made possible. In fact, during the post‐task debriefing, controls often vividly described how they would go about trying to construct some of the impossible semantic scenes.

For the constructive scenes, four patients and just one control described focussing closely on specific angles and intersection areas. The patients realised these individual parts were pertinent, but this realisation was clearly not sufficient, given their impairment on the task. In contrast, eleven control participants, but only two patients, described the discrimination process as considering the whole perspective or overall construction of the scene.

Despite the exploratory nature of these responses, it is interesting to note that even with similar accuracy performance on semantic scenes, the strategies reported by patients with hippocampal damage differed from those of control participants. That is, controls seemed to have a coherent, holistic and detailed internal model of both semantic and constructive scenes. By contrast, patients with hippocampal damage seemed to operate in a more constrained manner, sticking closely to the scene that was in front of their eyes, processing it in a fragmented fashion, with little indication of using an internal model of the scene.

## DISCUSSION

The current study sought to refine our understanding of hippocampal contributions to scene processing. In a novel task, we presented two types of impossibilities in scene images, semantic and constructive, that allowed us to hold general perceptual and mnemonic demands constant while isolating the need to construct spatially coherent scenes. We reasoned that deciding whether a scene is semantically possible or impossible depends on intact scene perception and scene comprehension, short‐term memory and semantic knowledge. The efficient discrimination of constructive possible and impossible scenes additionally depends on the ability to internally construct spatially coherent scenes. We found that patients with selective bilateral hippocampal damage had difficulty only in discriminating between possible and impossible constructive scenes, but not between possible and impossible semantic scenes. These results support the view that the contribution of the hippocampus to scene processing may be spatial‐constructive (Hassabis and Maguire, 2007; Maguire and Mullally, 2013; Zeidman and Maguire, 2016).

Our findings appear at odds with one study where it was reported that H.M., the amnesic patient first studied by Scoville and Milner (Scoville and Milner, 1957), detected fewer semantic impossibilities in a version of the children's game “What's wrong here?” compared with healthy control participants (MacKay and James, 2009). However, the stimuli used in that study were drawings of crowded scenes, each containing over ten semantic errors, such as a bird swimming in a fishbowl or a non‐functional door (Tallarico, 1991). Given that H.M.'s brain lesions extended well beyond the boundaries of the hippocampus (Annese et al., 2014), it is likely that his impaired semantic error detection was due to temporal neocortical damage. Moreover, intact hippocampal‐based scene construction would presumably be very useful in helping to detect multiple semantic errors in crowded scene images.

Here, we focussed specifically on semantic and constructive impossibilities within scenes, and our findings accord with a recent fMRI finding of increased hippocampal engagement during detection of impossible compared to possible constructive scenes (Douglas et al., 2016). By contrast, other studies have examined the neural substrates of possible and impossible objects. For example, evidence from an early positron emission tomography (PET) study suggested that the medial temporal lobes are involved in detecting the spatial coherence of objects (Schacter et al., 1995). However, the spatial resolution of this early finding precluded differentiation between different medial temporal lobe structures. We now know that the hippocampus itself is usually not involved in object processing (Lee et al., 2005b; Hassabis and Maguire, 2009; Barense et al., 2012; Mullally et al., 2012; Zeidman et al., 2014). Indeed, a patient with perirhinal cortex damage was impaired in discriminating between possible and impossible objects, whereas a patient with selective hippocampal damage performed similarly to healthy control participants (Lee and Rudebeck, 2010). In our study, we therefore selected realistic scene stimuli that we expected would require intact hippocampal functioning. Supporting the notion that we were indeed tapping scene processing, rather than object processing, controls described their strategies for the constructive scenes as constructing the entire scene in their imagination, rather than focussing on an object within the image. Interestingly, this global scene construction strategy was much less evident in the patients, which accords with other work showing that the attempts such patients make at scene construction are fragmented (Hassabis et al., 2007) and that they are biased towards local features in scenes (Aly et al., 2013).

Our results also question whether the hippocampus is involved in scene perception per se, since the detection of both semantic and constructive errors required intact scene perception or more general visual encoding of the scene images. From this perspective, other findings of hippocampal involvement in scene perception might also be interpreted as relying on the ability to construct a mental model of a scene (Lee et al., 2013, 2005b). That is, the tasks typically used to assess scene perception involve discrimination between highly similar scenes. In some cases, the scenes are presented from different viewpoints (Lee et al., 2013). Hence, one has to mentally rotate the scenes in order to compare them and detect the odd‐one‐out. This rotation process requires the mental construction of the scene; a task, we would argue, that requires an intact hippocampus.

Having said that, in healthy individuals scene perception and scene construction are probably very closely linked. We automatically model the scene we are currently perceiving (Mullally et al., 2012; Chadwick et al., 2013; Zeidman et al., 2015; Zeidman and Maguire, 2016). In fact, our control participants stated that they used scene construction processes automatically even during the search for semantic errors, despite this being unnecessary to achieve high accuracy on the task. Only by directly manipulating these processes and by testing patients with selective bilateral hippocampal damage could we start to pinpoint the hippocampal contribution to this intricate dialogue between scene perception and construction.

Another interpretation of the scene discrimination deficits seen in patients with hippocampal damage is that these patients are unable to compare two or more realistic scene images to each other because this exceeds the capacity of short‐term memory (Shrager et al., 2006; Kim et al., 2011; Kim et al., 2015). However, in our task each trial involved only one scene image which was always visible, circumventing the need to compare information across images, and therefore greatly minimising the mnemonic load. In addition, whatever general memory ability was necessary to perform this task (e.g., remembering the task instruction to decide if a scene was possible or impossible) was matched across the semantic and constructive conditions. We therefore believe that a purely mnemonic account of the selective deficit in detecting constructive impossibilities cannot explain our results.

A surprising observation from our study was that, despite no significant differences in accuracy or reaction times in the semantic scenes condition between control participants and patients, the strategies used by the two groups differed considerably. Controls described constructing vivid scenarios about how to make some of the impossible semantic scenes possible, whereas patients with hippocampal damage were much less likely to describe working flexibly with the scene images. It seems as if a functioning hippocampus readily engages and constructs internal models of scenes even though these are not always necessary for the task at hand. Although speculative, this observation is in line with previous research showing that hippocampal damage inhibits the creative and flexible use of internal representations of a wide variety of material (Duff et al., 2013; Rubin et al., 2014) but which, we suggest, typically involve creating spatially coherent scenes.

In conclusion, here we showed that patients with selective bilateral hippocampal damage have a specific difficulty discriminating between possible and impossible constructive scenes, indicating that the hippocampus has a particular and necessary role in constructing spatially coherent models of scenes regardless of semantic content. These findings refine our understanding of hippocampal function, and potentially its involvement in the higher order cognitive processes of perception and memory recall.
